# Environmental Stability Design of the Aerial Mapping Camera Based on Multi-Dimensional Compound Structure

**DOI:** 10.3390/s23094421

**Published:** 2023-04-30

**Authors:** Hong Yang, Guoqin Yuan, Jie Pan, DeYun Zhou

**Affiliations:** 1 School of Electronics and Information, Northwestern Polytechnical University, Xi’an 710129, China; yanghong@aircas.ac.cn (H.Y.); dyzhou@nwpu.edu.cn (D.Z.); 2 Aerospace Information Research Institute, Chinese Academy of Sciences, Beijing 100190, China; panjie@aircas.ac.cn; 3 Key Laboratory of Airborne Optical Imaging and Measurement, Fine Mechanics and Physics, Chinese Academy of Sciences, Changchun Institute of Optics, Changchun 130033, China

**Keywords:** aerial mapping camera, multi-dimensional compound structure, environmental stability technology

## Abstract

Environmental stability technology plays an important role in improving the adaptive range, image resolution and ensuring the stability of geometric parameters of aerial mapping camera. Traditional environmental stability methods directly implement active and passive thermal design to optical systems, which is easy to lead to radial temperature difference of optical components, and cannot eliminate the influence of pressure change. To solve the above problem, a method of environment stability design based on multi-dimensional structure is proposed. Firstly, the aerial mapping camera is designed as imaging system component (core) and sealing cylinder (periphery), and a sealed air insulation sandwich is formed between the two parts to realize the sealing design. A thermal interface is reserved outside the seal to avoid the radial thermal stress caused by direct heating of the optical parts, and a multi-dimensional Environmental stability structure is formed. Secondly, the core and the external thermal environment of aerial mapping camera in complex aviation environment are modeled and theoretically analyzed. Finally, the effectiveness and stability of the multi-dimensional structure method is verified by the thermal simulation and the flight. The results show that the thermal control power is 240 W, the thermal gradient of the optical system is less than 5 °C, the radial temperature difference is less than 0.5 °C. High quality image and ground measurement accuracy are obtained. Compared with tradition thermal control methods, the proposed method has the advantages of accuracy and low power consumption, which can effectively reduce the power consumption and difficulty of the thermal control.

## 1. Introduction

Aerial mapping camera is loaded in the general aviation carrier to complete the ground measurement, which has the advantages of high resolution, high measurement accuracy and flexibility. It is widely used in the earth observation, resource survey, navigation, disaster reduction and other fields [1,2,3,4]. Obtaining images with high resolution and high measurement accuracy is the ultimate goal of aerial mapping, but the complex and changeable aerial environment affects the performance and use range of the mapping cameras. Temperature and pressure are the important factors affecting the performance of aerial mapping camera [5,6,7]. First of all, the temperature change will lead to thermal stress on the aerial mapping optical components, reduce the shape of optical components, and the stability of the internal orientation element is difficult to predict, and eventually lead to the measurement accuracy and image quality reduction, secondly, such as pressure, temperature and environmental conditions of different working altitude can cause the change of glass material refractive index, make the optical system defocus, produce additional aberration, and the internal orientation element change, and eventually cause the decline of the aerial camera performance. Therefore, using the temperature and pressure environmental stability technology to maintain the temperature level of the aerial mapping camera optical system, eliminate the temperature gradient of the optical system, and maintain the pressure of the imaging system, it is particularly critical to improve the imaging quality and measurement accuracy of the aerial mapping camera. 

At present, the thermal control technology of space camera has been relatively mature [8,9,10,11,12], but there are few literatures on the thermal control of aerial mapping camera, especially the thermal control and pressure maintenance, so further research is needed. In general, the traditional calibration model usually adopted by aerial cameras is single-layer thermal control, that is, direct thermal control of the optical system, and the temperature level and temperature gradient of the optical system are controlled by means of conduction, convection and radiation, in order to obtain high-quality imaging images. Such as literature [13] on the aerial camera main optical system thermal control design, using the three traditional thermal control design method to realize that the R-C main optical system temperature difference is less than 5 °C thermal control effect, but the method of thermal control is a single layer thermal control method, which is not effective to isolate optical imaging system and the external environment, is failed to effectively eliminate the influence of pressure changes on optical performance. Literature [14] implements the thermal control design for the aviation photoelectric platform, and adopts active-passive combined thermal control technology to realize the temperature gradient of the primary and secondary mirrors in the optical system is no more than 5 °C; however, due to the direct conduction of heat transfer between the primary and secondary mirror components and the external environment, the thermal power consumption is high, about 370 W, and the stability of the orientation element is not mentioned in the implementation of environmental stability.

The aerial mapping camera has similarities in thermal control strategy and implementation, but it also has its own characteristics. Firstly, the temperature of the aerial mapping camera changes quickly, and it starts to work quickly from the ground to the working height, which has a large thermal impact on the optical imaging system. Secondly, when the aerial mapping camera is working in the troposphere or stratosphere of the atmosphere, it should consider the factors such as air convection heat transfer, and the environment is complex. Thirdly, the camera needs to be put into work immediately after takeoff, and the thermal control cycle is relatively short. The above characteristics determine that the design mode of the temperature and pressure environment stability of the aerial mapping camera is different from the traditional method. At present, in the literature, pneumatic sealing is rarely considered, which does not guarantee the stability of internal orientation elements, resulting in high thermal power consumption and radial thermal stress, which is not suitable for aerial mapping cameras that have high requirements on geometric elements.

Based on the detailed analysis of the thermal environment and the principle of heat transfer, a design method of multi-dimensional compound temperature and pressure is proposed for the aerial mapping camera. This method has the following advantages: first, the structure of the aviation camera is designed as two parts with the imaging system component and the sealing cylinder. Through the sealing design, a sealed air sandwich is formed between the sealing cylinder and the imaging component, and the above thermal control measures form the first dimension of thermal control and seal. Second, the heating film is pasted on the outside of the sealing cylinder, and the axial flow fan is installed on the inside of the same cavity as the optical imaging system, to eliminate the axial temperature difference, and avoid the radial temperature difference caused by direct heating optical components, forming a second dimension of thermal control. Third, the installation interface is reserved on the flange on both sides of the sealing cylinder for installing carbon fiber materials with poor thermal conductivity, and coating heat insulation foam on the outside of the carbon fiber to isolate the external environment and form the third dimension of heat control. After completing the stability of the above-mentioned multi-dimensional compound environment stability design, the thermal simulation model of the optical machine structure is established, and the thermal simulation analysis is conducted to verify the effectiveness of the present method. Finally, based on the actual flight test, the high-quality image and high-precision measurement are obtained.

The rest of the paper is organized as follows. The Aerial mapping camera optical system is described in Section 2. The thermal environment analysis is given in Section 3. The proposed method is described in Section 4. Results from validating the method both in simulation and on real data are presented in Section 5 and Section 6. Finally, Section 7 presents the conclusions.

## 2. Aerial Mapping Camera

**Figure 1:**
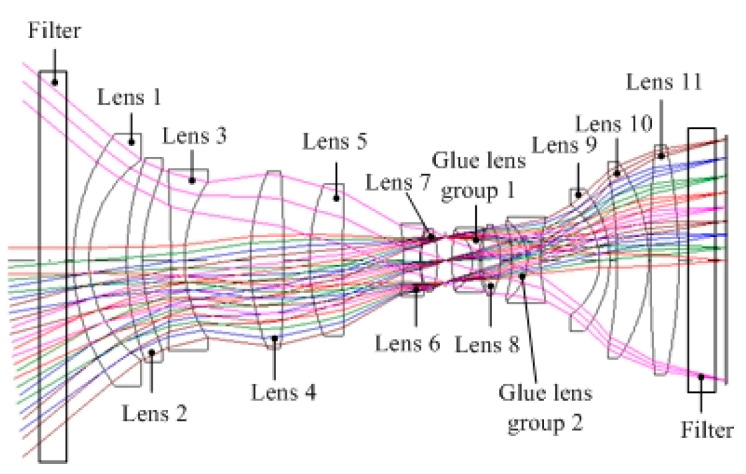
Schematic diagram of optical system of aerial mapping camera.

The aerial mapping camera uses a transmission optical system, as shown in Figure 1. There are 17 lenses with an axial size of over 500 mm. The aeronautical mapping camera has a focal length of 130 mm and a field of view of 79 degrees. The comparison of the camera and the international advanced ADS100 mapping camera is shown in Table 1. It can be seen that the technical indicator of thermal control objects are higher than ADS100, which need higher requirements for thermal control. In order to realize high-precision mapping, it is not suitable to set up a focusing mechanism, and it is necessary to seal it out to eliminate the influence of external pressure changes. The traditional single-layer thermal control method is difficult to meet the requirements of temperature and pressure stability.

The aerial mapping camera optical system is shown in Figure 1. In order to improve the transmittance of the system, the surface of the optical lens is coated with multi-layer reduced reflection film to ensure that the transmittance of each surface is more than 99%. Considering the outer surface of the lens, the adhesive surface and the absorption of the lenses, the transmittance of the whole system is more than 61%. The design method of the image space telocentric optical system is adopted to ensure that the light incident to the focal surface is parallel to the main light. The incidence angle of the image main light field of view is less than 10°, which ensures that the image point can still be maintained in the original position while little out-of-focus happened, so as to ensure the measurement accuracy.

According to the thermal analysis results of the optical system, the working temperature range of the mapping camera is 20 ± 20 °C, the axial temperature difference of the system is less than 5 °C, the radial temperature difference is less than 1 °C, and the pressure is constant.

## 3. Thermal Environment Analysis

Thermal environment is the boundary condition and design input for the stability design of the multi-dimensional compound environment. Analyzing the working thermal environment is the necessary condition for implementing the stability design. Thermal environment is mainly divided into internal thermal environment and external thermal environment. The internal thermal environment refers to the initial temperature of the camera and the internal heat source; the external thermal environment mainly refers to the atmospheric environment (altitude, temperature, density, pressure) and flight speed [15].

### 3.1. The Internal Thermal Environment

The heat exchange between the imaging system and the sealing cylinder is mainly heat conduction and heat radiation.

According to the law of thermodynamics, the heat conduction formula is as follows:

(1) $$\phi =\frac{∆T}{R}$$

In Equation (1), R is the thermal resistance. Specifically, R can be divided into two parts: conduction thermal resistance $R_{1}$ and contact thermal resistance $R_{2}$; Δ*T* is the temperature difference [16] between the imaging system and the seal cylinder.

$R_{1}$ is used to indicate the resistance during the heat transfer process. The expression is as follows:

(2) $$R_{1}=\frac{\delta }{\zeta A}$$

In Equation (2), ζ(W·m^−1^·K^−1^) is the thermal conductivity, which is determined by the thermal conductive material; δ is the contact surface thickness. A (m^2^) is the area of the contact surface area. After the material is selected, the thermal conductivity ζ is determined accordingly, and the thermal resistance is directly proportional to the thickness of the contact surface and inversely proportional to the area of the contact surface. Due to the influence of surface roughness and other factors, the imaging system and the binding surface of the sealing cylinder cannot completely fit. Generally, there is micro irness, which leads to the air gap layer between the two, which will lead to additional thermal resistance. This value is related to the material properties, surface roughness, contact pressure and other factors of the contact surface. The expression is as follows:

(3) $$R_{2}=\frac{1}{K_{t}A}$$

where $K_{t}$ (W·m^−2^·K^−1^) is the contact thermal resistance coefficient.

The calculation formula for thermal radiation is:

(4) $$\Phi_{in2}=\varepsilon_{s}A_{2}\sigma \left({T_{1}}^{4}-{T_{2}}^{4}\right)$$

where $\varepsilon_{s}$ is the emit rate of the system; σ is the Stefan-Boltzmann constant, $A_{2}$(m^2^) is the radiation surface area; $T_{1}$ is the internal side temperature of the outer chamber, and $T_{2}$ is the surface temperature of the optical machine structure. The internal heat source will have a great impact on the temperature distribution of the camera, and its uneven distribution is an important factor to produce the temperature gradient. The nonuniformity of heat radiation can be eliminated by the axial flow fan.

### 3.2. The External Thermal Environment

Aerial mapping cameras are usually mounted on the belly of the carrier, the upper part is inside the carrier, and the lower part is directly exposed in the airborne environment. When the typical flight altitude is 800~2000 m and 2000 m, the typical flight speed is 240 km/h, which is slow, so only the effects of radiation and convection are considered.

Considering the actual situation of the camera operation during the flight, the following heat exchange between the camera and the surrounding environment: the heat conduction exchange between the contact surface of the carrier, the heat convection with the external atmosphere, and the heat exchange with the ground infrared radiation.

#### 3.2.1. Convective Heat Exchange

During the forward flight of the carrier, there is heat conduction exchange between the camera and the external atmosphere through convection, and the model can be expressed as a fluid swept flat wall model.

According to the convective heat transfer theory of boundary layer, the heat transfer coefficient at laminar and turbulent flow can be determined by the Nusel criterion:

(5) $$N_{u}=\frac{h_{Lx}x}{\zeta }=0.332{R_{e}}^{\frac{1}{2}}{P_{r}}^{\frac{1}{2}},\left(R_{e}<5\times 10^{5},0.6<P_{r}<50,laminar\;conditions\right)$$

(6) $$N_{u}=\frac{h_{tx}x}{\zeta }=0.0296{R_{e}}^{\frac{4}{5}}{P_{r}}^{\frac{1}{3}},\left(R_{e}<5\times 10^{5},0.6<P_{r}<50,turbulent\;conditions\right)$$

where $h_{Lx}$,$h_{tx}$ ($W\cdot m^{-2}\cdot K^{-1}$ ) is the convective heat transfer coefficient; ζ is the atmospheric thermal conductivity; $P_{r}$ is the Plandor number; $R_{e}$ is the Reynolds number, $R_{e}=\frac{\rho vl}{\mu }$, where ρ ($Kg/m^{3}$) is the density of the fluid; *v* (m/s) is the relative velocity; *l* (m) is the convection length; μ is the viscosity coefficient.

In the actual operation of the camera, the turbulent boundary layer in the atmospheric vertical sweep flat wall model generally occurs at the back of the flat wall, while the front laminar boundary layer remains unchanged. In the flat wall model, the average convective heat transfer coefficient of the surface can be expressed as:

(7) $$h=\frac{1}{l}\left[\int_{0}^{x_{c}}h_{Lx}d_{x}+\int_{x_{c}}^{l}h_{tx}d_{x}\right]=0.037\frac{\zeta }{l}\left({R_{e}}^{0.8}-23500\right){P_{r}}^{\frac{1}{3}}$$

#### 3.2.2. Heat Radiation

Ground infrared radiation is related to surface temperature and surface emissivity. In general, taking the average solar constant $\overset{-}{S_{0}}=1353\;W/m^{2}$, the average infrared thermal radiation density of the earth’s surface can be expressed as:

(8) $$q_{e}=\varepsilon \overset{-}{S_{0}}=0.35\times 1353=473.6\;W/m^{2}$$

where ε is the surface emissivity. When infrared thermal radiation from the earth’s surface enters the atmosphere, it is reflected by the terrain and by clouds besides being absorbed by the atmosphere. Generally, if the terrain surface is treated as a black surface, the average solar shortwave mean irradiance can be calculated as follows:

(9) $$q_{r}=\frac{\overset{-}{S_{0}}\left(1-\varepsilon \right)}{4}=220\;W/m^{2}$$

## 4. Multi-Dimensional Compound Stability Design

**Figure 2:**
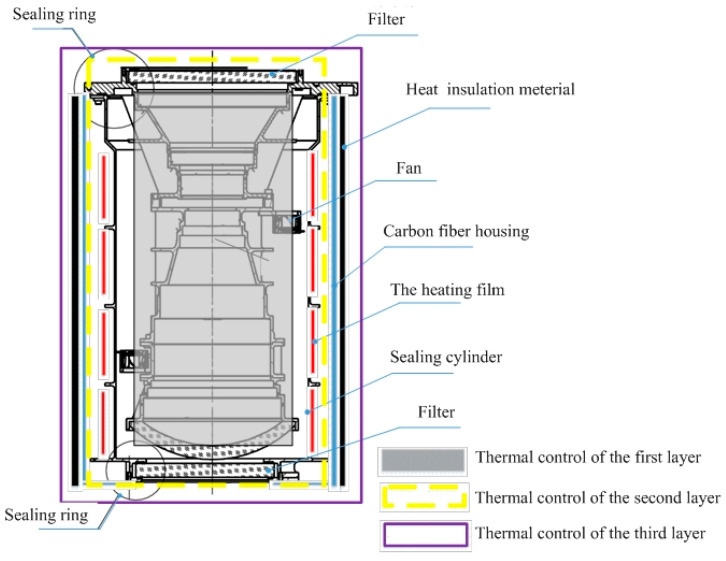
Schematic diagram of Environmental stability design of aerial mapping camera.

According to the environmental requirements of the optical system, in order to ensure the stability of the internal orientation element, the multilayer structure stability design method is adopted. The optical lens is designed as two parts: sealing cylinder and imaging system, as shown in Figure 2. The imaging system is a component of the imaging optical system. The whole optical imaging system is installed in the sealing cylinder through the front filter and filter to realize the sealing design. The sealing cylinder adopts the aluminum-magnesium alloy material with low density and good thermal conductivity to realize the air-tight design. The heating film is pasted on the outside of the sealing cylinder, and the optical components are heated by thermal radiation to avoid the radial temperature difference caused by the direct heating of the optical components. The axial flow fan installed inside the sealing cylinder can effectively eliminate the axial temperature difference of the optical system. Seal cylinder outside reserved the third dimension thermal control interface, the third dimension thermal control choose carbon fiber cover with insulation foam, realize the optical system and external environment effective thermal isolation, through multi-dimensional compound design way to avoid the direct heating of optical components thermal stress, avoid the pressure focus, ensure the geometric stability, finally guarantee high quality imaging and high precision measurement to the ground.

Multi-dimensional compound environment stability design method through the aerial mapping camera is divided into imaging system, sealing cylinder, cover, and other parts. The imaging system sealed and isolated in the outside environment, then active and passive thermal control is implemented, eventually a cover, sealing cylinder, air interlayer, imaging system and other multi-dimensional compound structure is formed. The active and passive thermal control and other measures were used to control the temperature and pressure, finally to meet the requirements of stable imaging and mapping.

### 4.1. Heat Insulation Layer

The heat insulation layer can reduce the heat exchange between the aerial mapping camera and the external environment, and reduce the pressure and power consumption of the active thermal control. In order to adapt to the complex environment, the heat insulation layer should have the characteristics of low thermal conductivity, low temperature and low pressure resistance, black, good flame retardant performance and so on [17].

After experimental comparison, the microporous polyurethane rubber insulation material is selected. In the actual selection process, the thickness of the insulation layer should be determined according to the thermal resistance and weight of the material.

Material thermal resistance *R_m_* can be expressed as:

(10) $$R_{m}=\frac{d}{\lambda_{m}\cdot A_{m}}$$

where d (m) is the thickness of microporous polyurethane rubber; $\lambda_{m}$ is the thermal conductivity of microporous polyurethane rubber, and $A_{m}$ ($m^{2}$) is the area of coated microporous polyurethane rubber.

The larger the thickness of microporous polyurethane rubber, the higher the thermal resistance, the better the heat insulation effect. But at the same time, the larger weight is not conducive to the lightweight design of the whole machine, which be considered between the weight and thermal resistance. In this paper, 15 mm thick insulation material is selected, and the sealing cylinder is covered as shown in Figure 2.

### 4.2. Thermal Control of the Optical Imaging System

Optical imaging system is the core of the whole aerial mapping camera, which is easily sensitive to temperature and has high requirements for thermal control. After thermal and optical property analysis, the lens temperature level of the whole optical system is 20 °C ± 20 °C; the axial temperature difference shall not exceed 5 °C and the radial temperature difference shall not exceed 0.5 °C.

The polyimide heating film is arranged in the sealing cylinder, and the partition thermal control is conducted. When the temperature sensor detects that the temperature change of the optical system exceeds the threshold value, the heating film begins to energize and work, and ensure that the optical system is in the set temperature range through active thermal control.

**Figure 3:**
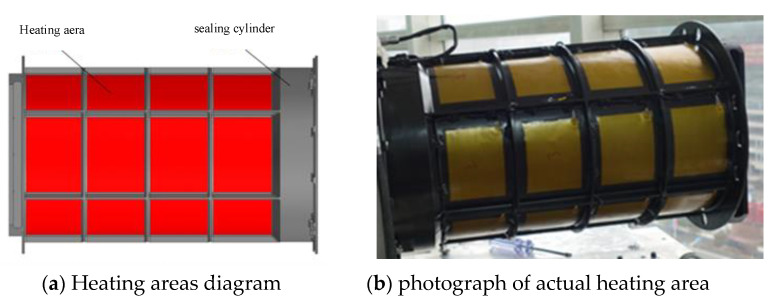
Schematic and physical diagram of heating area of sealing cylinder.

Limited by the optical machine structure, generally, the temperature sensor cannot be directly bonded to the lens, so the temperature sensor is evenly arranged on the outer wall of the lens cylinder corresponding to the lens position. Figure 3 is a schematic diagram of the heating area on the outside of the sealing cylinder. Sixteen heating areas are arranged symmetrically, and the power consumption of each heating area is 15 W, for a total of 240 W.

## 5. Thermal Simulation Analysis

**Figure 4:**
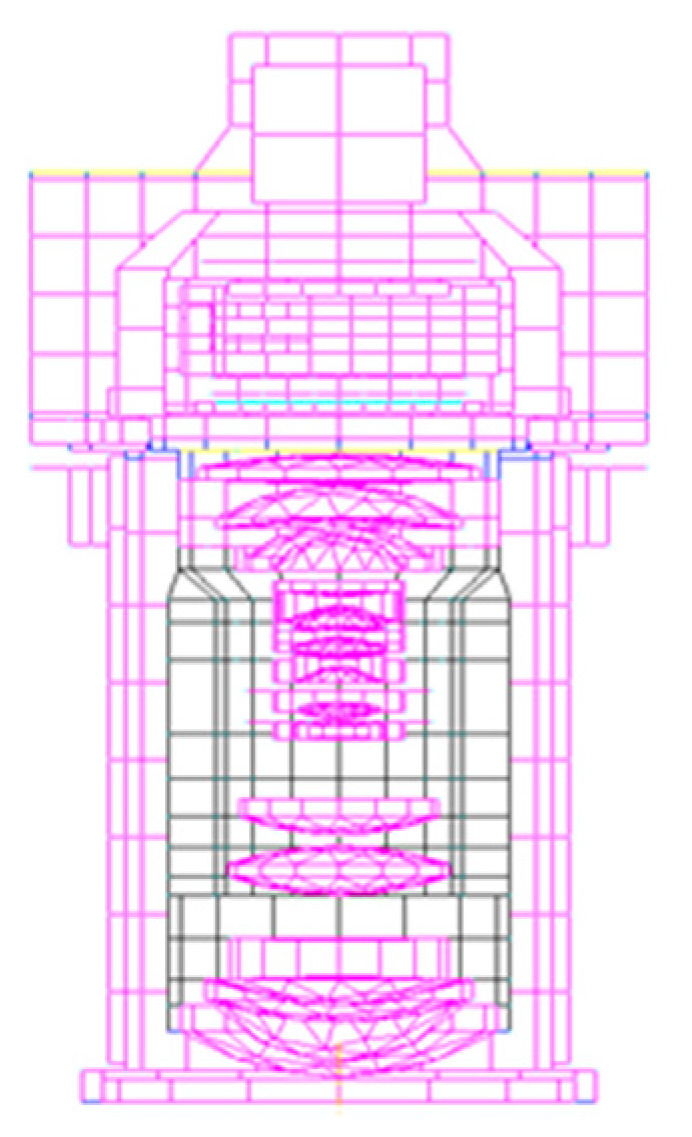
Thermal analysis model of aerial mapping camera.

A thermal model of the aerial mapping camera was established using the thermal analysis software, and the finite element model is shown in Figure 4. Considering the actual working scenario, two typical working conditions are set:

1. The camera and ambient temperature is 20 °C, and the flight altitude is 2000 m;
2. The camera and ambient temperature is −20 °C, and the flight altitude is 2000 m.

From the internal and external thermal environment analyzed in Section 3. The boundary constraints are imposed on the finite-element thermal model which was calculated.

### 5.1. Overall Thermal Control Analysis

**Figure 5:**
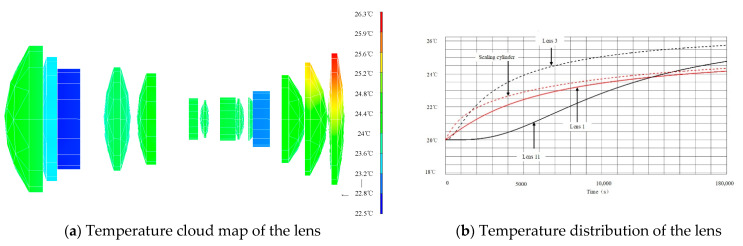
Temperature of lens after 6 h of work under condition 1.

Figure 5 shows the temperature cloud map of the lens and the whole machine after 6 h worked. At this time, the lens temperature is between 22.5 °C and 26.3 °C, the maximum temperature difference is 3.8 °C, the deviation from the initial temperature (20 °C) is small, and the lens temperature gradient is small; the temperature difference of the imaging system is small.

**Figure 6:**
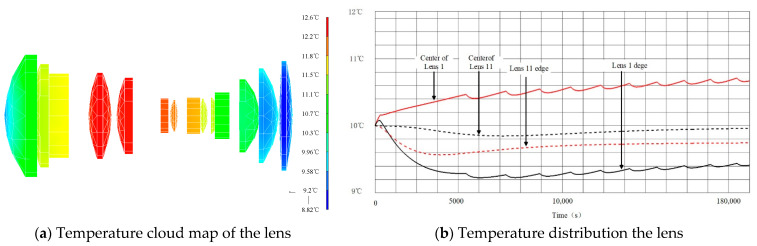
Temperature cloud image of lens after 6 h of work under condition 2.

Figure 6 shows the cloud map of the lens and the whole machine temperature after the camera works for 6 h. At this time, the lens temperature is between 8.82 °C; and 12.6 °C, the maximum temperature difference is 3.78 °C, and the lens temperature gradient is small; the temperature difference of the imaging system is small.

From the above analysis results, it can be seen that under the low temperature and low temperature environment, the temperature level of the optical system lens can be maintained at 20 ± 2.5 °C, and the temperature difference of the optical machine structure is relatively small, which meets the thermal control requirements of the optical system.

### 5.2. Data Analysis

From the comparison of the above data, the following conclusions are obtained:

3. With the multi-dimensional compound stability design implementation, the maximum temperature difference between lenses is not greater than 5 °C, the temperature level of the optical system lens is improved, the temperature gradient is reduce, and the temperature level hold time is extended, the external environment effect on the internal optical system of camera is effectively weakened. Camera overall cooling speed is slow, and the uniformity of temperature is conductive to the camera;
4. The multi-dimensional compound stability design ensures the pressure and temperature of the camera, and eliminates the impact of the external environmental pressure change on the imaging performance of the mapping camera;
5. Multi-dimensional compound stability design can greatly improve the temperature level of the camera and control the temperature gradient of the optical system with good effect.

## 6. Flight Test

**Figure 7:**
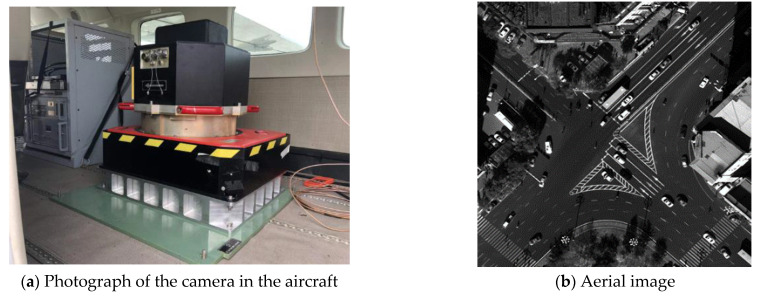
Photograph of the camera in the aircraft and the acquisition image.

To verify the effectiveness of the environmental stability design, flight tests were performed. Flight test is the most direct and effective way to check the effect of environmental stability design of the aerial mapping camera. It can avoid errors caused by simulated calculation and thermal coupling, and can truly reflect the temperature distribution and mapping results of aerial mapping cameras. While the flight altitude is 2 km, the atmospheric pressure drops by 7% compared with the ground when working. The air-tight design measures adopted by the camera eliminate the influence of the atmospheric pressure change and ensure the constant internal pressure of the optical system in the process of working. During the test, the camera worked properly, and high-quality aerial images were obtained, as shown in Figure 7.

**Figure 8:**
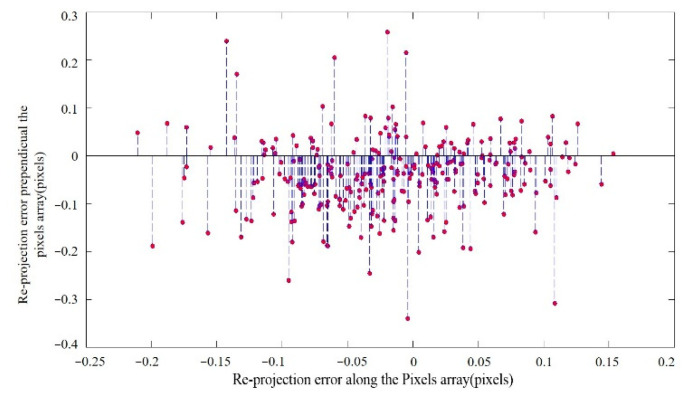
Reprojection error of check points.

Re-projection error is used to evaluate the performance of the new system. Taking the measured 3D points projected in the cameras with the camera calibration parameters again, obtaining new points in the camera. Then calculate the pixel value between the original points and the pixel value difference between the actual point and the calculated projection point is the “re-projected” ones [18]. The ground checkpoint re-projection error is shown in Figure 8, according to which we found the re-projection is better than 1 pixel. The results indicate that the camera imaging quality is good, and the internal orientation elements are stable.

From the flight experiment result, we can see that the proposed design method is effective, which ensures the constant pressure, and effectively controls the temperature level and temperature gradient of the optical system, and ensures the stable and clear aerial image and high measurement accuracy of the mapping camera.

## 7. Conclusions

In view of the traditional design method of single layer structure, which is difficult to guarantee the imaging quality and the stability of the orientation element, a multi-dimensional compound environment stability structure design method for aerial mapping camera is proposed, so that the lens temperature in the optical system has a good buffer, and effectively reduce the active thermal control power consumption. Another contribution of this paper is to realize the sealing of the imaging system in the thermal control implementation, obtaining high quality aerial images and measurement precision. The results of thermal simulation analysis and thermal test show that the thermal control power consumption of the system is 240 W, and the temperature gradient of the optical system does not exceed 5 °C, which meets the requirements of thermal control design. Therefore, the design method of multi-dimensional compound environment stability structure is simple, accurate and easy to implement, which can effectively reduce the power consumption of thermal control. After actual flight verification, this method obtains high quality aerial image and measurement accuracy.

## Figures and Tables

**Table 1:** The research object VS. ADS100.

Indicators	Aerial Mapping Camera of This Paper	ADS100
Focal length	130 mm	62.5 mm
Field of View	79°	77.3°
Imaging plane size	107.2 mm	49.98 mm
dimension	Φ500 mm X740 mm	Φ390 mm X670 mm
weight	60 Kg	50 Kg

## Data Availability

Not applicable.
